# Impairment in renal medulla development underlies salt wasting in Clc-k2 channel deficiency

**DOI:** 10.1172/jci.insight.151039

**Published:** 2021-10-22

**Authors:** Meng-Hsuan Lin, Jen-Chi Chen, Xuejiao Tian, Chia-Ming Lee, I-Shing Yu, Yi-Fen Lo, Shinichi Uchida, Chou-Long Huang, Bi-Chang Chen, Chih-Jen Cheng

**Affiliations:** 1 Division of Nephrology, Department of Medicine, Tri-Service General Hospital, and; 2 Graduate Institute of Life Sciences, National Defense Medical Center, Taipei, Taiwan.; 3 Brain Research Center, National Tsing Hua University, Hsinchu, Taiwan.; 4 Research Center for Applied Sciences, Academia Sinica, Taipei, Taiwan.; 5 Laboratory Animal Center, College of Medicine, National Taiwan University, Taipei, Taiwan.; 6 Department of Nephrology, Graduate School of Medical and Dental Sciences, Tokyo Medical and Dental University, Tokyo, Japan.; 7 Division of Nephrology, Department of Internal Medicine, Carver College of Medicine, University of Iowa, Iowa City, Iowa, USA.

**Keywords:** Nephrology, Chloride channels, Chronic kidney disease

## Abstract

The prevailing view is that the ClC-Ka chloride channel (mouse Clc-k1) functions in the thin ascending limb to control urine concentration, whereas the ClC-Kb channel (mouse Clc-k2) functions in the thick ascending limb (TAL) to control salt reabsorption. Mutations of ClC-Kb cause classic Bartter syndrome, characterized by renal salt wasting, with perinatal to adolescent onset. We studied the roles of Clc-k channels in perinatal mouse kidneys using constitutive or inducible kidney-specific gene ablation and 2D and advanced 3D imaging of optically cleared kidneys. We show that Clc-k1 and Clc-k2 were broadly expressed and colocalized in perinatal kidneys. Deletion of Clc-k1 and Clc-k2 revealed that both participated in NKCC2- and NCC-mediated NaCl reabsorption in neonatal kidneys. Embryonic deletion of Clc-k2 caused tubular injury and impaired renal medulla and TAL development. Inducible deletion of Clc-k2 beginning after medulla maturation produced mild salt wasting resulting from reduced NCC activity. Thus, both Clc-k1 and Clc-k2 contributed to salt reabsorption in TAL and distal convoluted tubule (DCT) in neonates, potentially explaining the less-severe phenotypes in classic Bartter syndrome. As opposed to the current understanding that salt wasting in adult patients with Bartter syndrome is due to Clc-k2 deficiency in adult TAL, our results suggest that it originates mainly from defects occurring in the medulla and TAL during development.

## Introduction

Chloride ion (Cl^–^) is the major anion providing charge balance to sodium ion (Na^+^). Cl^–^ transport across the renal tubule is thus vital to renal NaCl reabsorption. The ClC-type chloride channels ClC-Ka and ClC-Kb are expressed in the ascending limbs and distal nephrons of human kidneys, and human mutations in these channels have been associated with disorders of salt and water homeostasis (1–4).

Bartter syndrome, a hereditary renal tubulopathy affecting NaCl reabsorption in the thick ascending limb (TAL) of the loop of Henle, is divided into antenatal and classic types based on the severity of perinatal presentation. Antenatal Bartter syndrome is caused by mutations in either the sodium-potassium-chloride cotransporter type 2 (NKCC2) or the renal outer medullary potassium (ROMK) channel, both of which are critical for NaCl entry through the apical membrane of TAL. In contrast, classic Bartter syndrome, caused by loss-of-function mutations in ClC-Kb in the basolateral membrane of TAL, has more-variable phenotypes, ranging from perinatal disease to adolescent-onset salt wasting (5). A recent report suggests that genotype accounts for phenotype variabilities (6, 7), but this hypothesis does not explain the phenotypic variability observed commonly among siblings carrying the same ClC-Kb mutation (8).

In contrast to ClC-Kb, the precise role of ClC-Ka in salt and water transport in humans is less clear. Individuals with presumed gain-of-function single nucleotide polymorphisms of ClC-Ka are reportedly more susceptible to volume expansion and salt-sensitive hypertension, suggesting its role in renal salt handling (3, 4). It is reported that a human infant with rare simultaneous ClC-Ka and ClC-Kb mutations required more salt and potassium supplementation than is usual in classic Bartter syndrome (9). Altogether, these reports raise the possibility that ClC-Ka is also involved in NaCl reabsorption.

Studies on Clc-k1 and Clc-k2, rodent orthologs of human ClC-Ka and ClC-Kb, have provided information pertaining to the respective function of the 2 channels (10–12). Clc-k1 was found mainly on the apical and basolateral membranes of the thin ascending limb (tAL) (13), while Clc-k2 was most abundant on the basolateral membrane of TAL, distal convoluted tubule (DCT), and intercalated cells of the collecting duct (14). Other studies also show that Clc-k1 and Clc-k2 are differentially expressed and barely colocalized in mouse kidneys (14, 15). A recent patch-clamp experiment reaffirmed that Clc-k2 is the dominant Cl^–^ channel in the distal nephron, without evidence of much Clc-k1 therein (15). Clc-k1–null mice manifested nephrogenic diabetes insipidus (NDI), whereas Clc-k2–null mice recapitulated classic Bartter syndrome (15–17). Overall, these findings have led to the current notion that Clc-k1 functions in the tAL to control urinary concentration and Clc-k2 functions in TAL to control NaCl reabsorption.

The latest transcriptome analysis of individually dissected adult mouse renal tubules, however, indicates broader and more-abundant Clc-k1 expression in the distal nephron than previously suggested, implying a significant functional overlap between Clc-k1 and Clc-k2 (18). While it has been suggested that ClC-Ka may compensate for ClC-Kb deficiency and ameliorate the phenotype of classic Bartter syndrome, direct supporting evidence has been lacking.

We set out to better understand the physiological role of Clc-k1 and Clc-k2 in the perinatal kidney, and we hypothesized that the developmental expression of Clc-k1 and Clc-k2 and their relative roles may shed light on the phenotypical variabilities and early pathogenesis of classic Bartter syndrome in some patients. We employed constitutive and inducible renal tubule–specific *Clcnk* gene–deleted mice and analytic tools that included clearance studies and advanced 3D imaging of optically cleared kidneys. We report that both Clc-k1 and Clc-k2 were broadly expressed in developing kidneys and contributed to salt reabsorption. Moreover, perinatal Clc-k2 activity was vital for medulla development, which critically affected the ability of kidneys to reabsorb NaCl in adulthood.

## Results

### Clc-k1 and Clc-k2 expression in developing and developed mouse kidneys.

**Figure 1:**
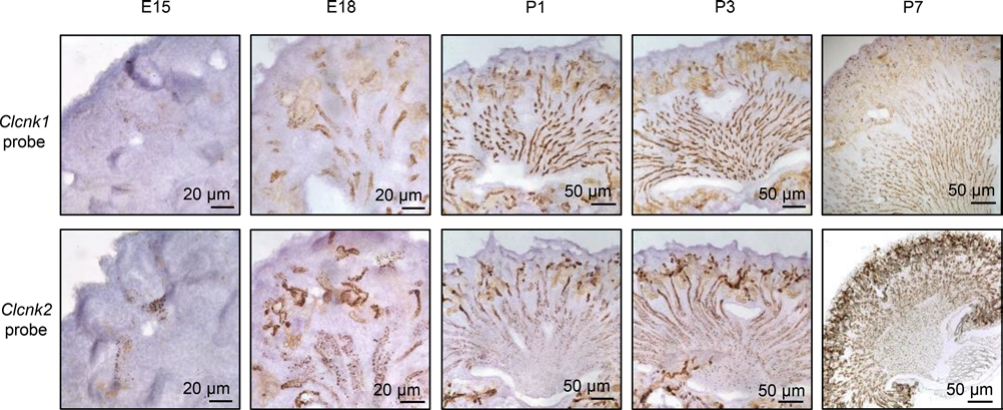
Spatiotemporal expression of Clc-k1 and Clc-k2 in the developing mouse kidney. Perinatal mouse kidneys (E15 to P7) hybridized with riboprobes specific to mouse *Clcnk1* and *Clcnk2* mRNA. E13 kidneys are absent of *Clcnk1* or *Clcnk2* mRNA (data not shown here; see Supplemental Figure 1 for the specificity of *Clcnk* riboprobes). Scale bars: 20 μm (E), 50 μm (P).

**Figure 2:**
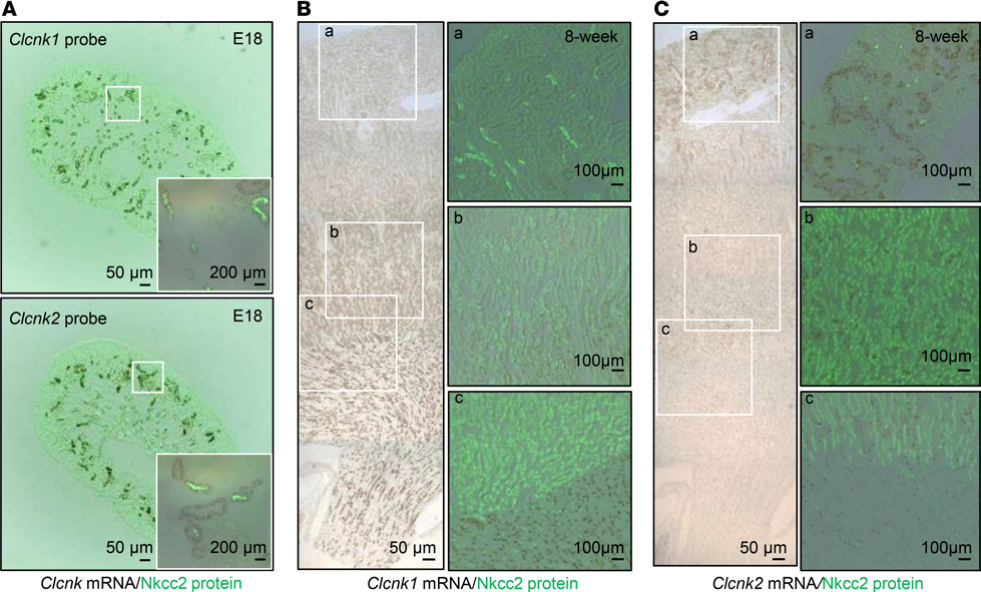
Localization of *Clcnk1* and *Clcnk2* mRNA transcripts in embryonic and adult kidneys. (**A**) In situ hybridization revealed mRNA signals of *Clcnk1* and *Clcnk2* in E18 embryonic kidneys. Both *Clcnk1* and *Clcnk2* mRNA were detected in the renal cortex and medulla, including TAL, where *Clcnk* mRNA signals encircled the green fluorescence of Nkcc2, suggesting the colocalization between Clc-k1 and Clc-k2 in the developing loop of Henle. (**B** and **C**) Superimposed Nkcc2 IFC staining and in situ hybridization of *Clcnk1* and *Clcnk2* mRNAs depict the distribution of Clc-k1 and Clc-k2 along the Nkcc2-positive TAL in 8-week-old adult mouse kidneys. Areas inside the white squares were magnified (a, cortex; b, outer medulla; c, boundary between outer and inner medulla). Scale bars: 50 μm, insets 200 μm (**A**); 50 μm, insets 100 μm (**B** and **C**).

We examined expression of *Clcnk1* and *Clcnk2* mRNA transcripts in developing mouse kidneys from E13 to P7 using gene-specific riboprobes (*Clcnk1* and *Clcnk2*, respectively; for probe validation, see Supplemental Figure 1; supplemental material available online with this article; https://doi.org/10.1172/jci.insight.151039DS1). Clc-k2 appeared first in the nephrogenic zone on E15, whereas Clc-k1 appeared around E18 (Figure 1). In E18 embryonic kidneys, both *Clcnk1* and *Clcnk2* mRNAs became more diffusely expressed in the renal cortex and medulla, including in the Nkcc2-positive loop of Henle (Figure 2A). On P1 and P3, *Clcnk2* transcript levels increased in the cortex, while they decreased in the medulla; in contrast, *Clcnk1* transcripts were gradually confined within the developing medulla (Figure 1). By P7, Clc-k1 and Clc-k2 became preferentially localized to the medulla and cortex, respectively. Barttin is an accessory protein of Clc-k channels. Immunohistochemistry revealed that barttin was diffusely expressed in the cortex and medulla of P1–P7 kidneys (Supplemental Figure 2). The results indicate that the differential distribution of Clc-k1 and Clc-k2 in P1–P7 kidneys is not governed by barttin.

**Figure 3:**
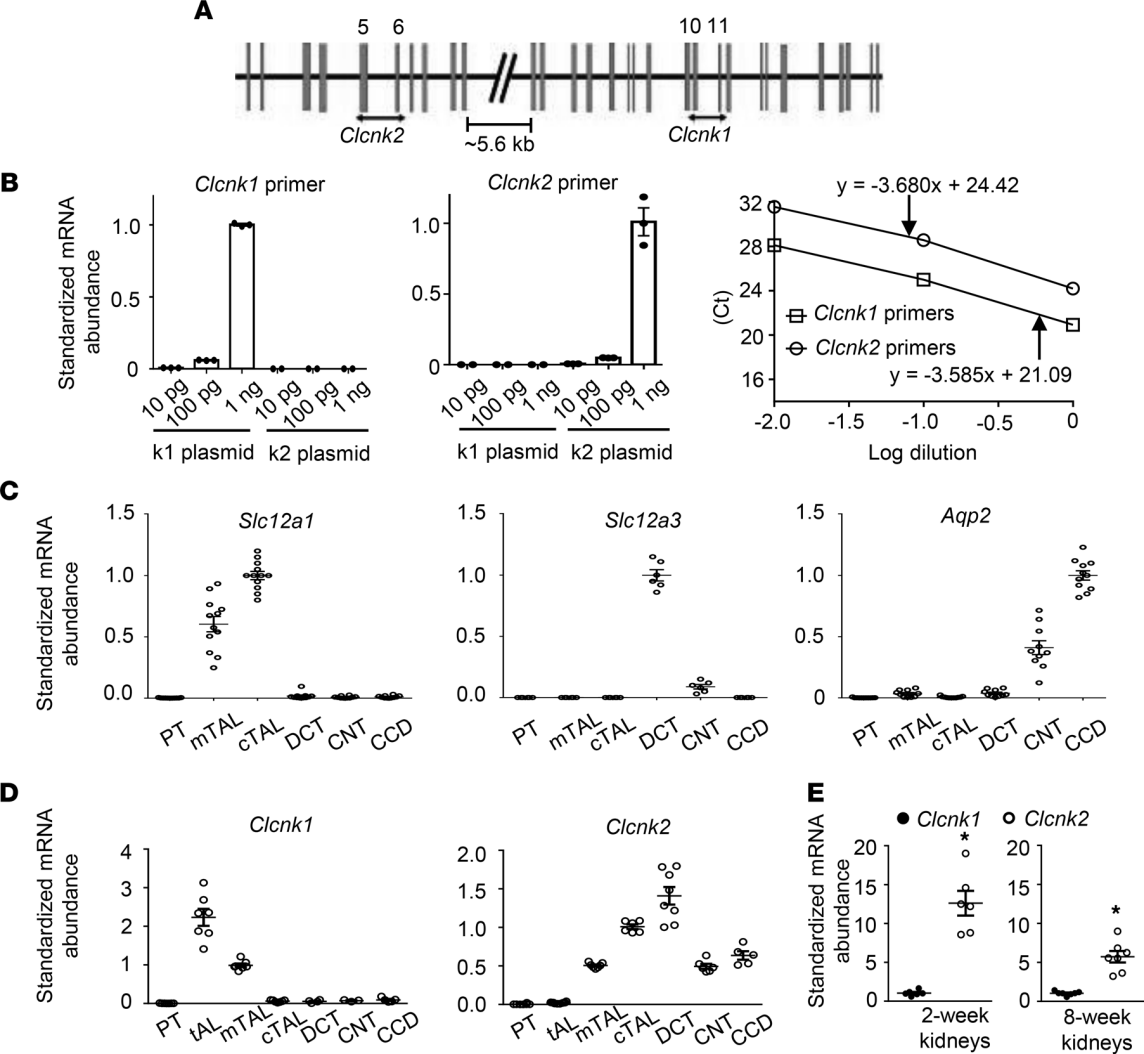
Quantitative measurements of *Clcnk1* and *Clcnk2* mRNAs in each WT renal tubular segment and whole kidneys. (**A**) Schematic representation of *Clcnk1* and *Clcnk2* exons shows the positions of primers (double-headed arrows) used for RT-PCR analysis of Clc-k1 and Clc-k2 in this study. The mouse *Clcnk1* and *Clcnk2* genes are closely adjacent to each other in the same chromosome (chromosome 4), with an approximately 5.6 kb genomic DNA fragment separating the 2 genes (**B**) Fixed amounts (10 pg, 100 pg, 1 ng) of mouse Clc-k1 and Clc-k2 plasmids were used to test the primers’ efficacy and specificity. The relative abundance of Clc-k1 or Clc-k2 was normalized to the mean of 3 replicas of 1 ng reaction. The measured threshold cycle was plotted against the log of the dilution. The results showed similar efficiencies (slope of the linear equation) of the Clc-k1 and Clc-k2 RT-PCR assays. (**C**) Tubule-specific markers (*Slc12a1* to TAL, *Slc12a3* to DCT, *Aqp2* to CCD) were measured to confirm the purity of the dissected tubules. The average amount of target genes in the highest-expressed tubular segment (cortical TAL [cTAL] for *Slc12a1*, DCT for *Slc12a3*, CCD for *Apq2*) was set as 1. The renal tubules were isolated from WT mice 6~8 weeks of age (*n* ≥ 6 for each renal tubule segment). (**D**) Standardized mRNA levels of *Clcnk1* and *Clcnk2* in each tubular segment. *Clcnk1* and *Clcnk2* in each sample were standardized to the mRNA level of its own housekeeping *Gapdh* gene. The average amount of *Clcnk1* in mTAL and *Clcnk2* in cTAL was set as 1. The renal tubules were isolated from WT mice 6~8 weeks of age (*n* ≥ 6 for each renal tubule segment). (**E**) Standardized mRNA levels of *Clcnk1* and *Clcnk2* in 2- and 8-week-old mouse whole-kidney lysates. The mean of *Clcnk1* mRNA level was set as 1 (*n* ≥ 6 for each group). The comparison between *Clcnk1* and *Clcnk2* was performed by unpaired *t* test. *P < 0.05 between *Clc-k1* and *Clc-k2*. PT, proximal tubule; CNT, connecting tubule).

In 8-week-old adult kidneys, *Clcnk1* and *Clcnk2* mRNAs were found mostly in the medulla and cortex, respectively, with only a small amount of Clc-k1 and Clc-k2 colocalized in the outer medulla (Figure 2, B and C). We further investigated the differential expression of Clc-k1 and Clc-k2 in renal tubules individually isolated from 8-week-old WT mice. The validity of quantitative PCR and identity of isolated tubular segments were authenticated (Figure 3, A–C). As shown in Figure 3D, Clc-k1 was predominantly expressed in tAL, with a reduced amount in medullary TAL (mTAL). Clc-k2 was more widely expressed in tubules from mTAL to cortical collecting duct (CCD), with the most abundance in DCT. Thus, *Clcnk1* and *Clcnk2* transcripts were restricted to tubules in the medulla and cortex, respectively, with a singular overlap in mTAL. The notion was further supported by IFC staining (see below). Analysis of relative transcript abundance revealed that Clc-k2 levels were approximately 12-fold greater than Clc-k1 levels in 2-week-old kidneys, a difference that decreased to approximately 6-fold in 8-week-old kidneys, likely due to the development of tAL and renal medulla (Figure 3E).

### Dual deletion of Clc-k1 and Clc-k2 amplifies renal salt wasting and hypovolemia.

**Figure 4:**
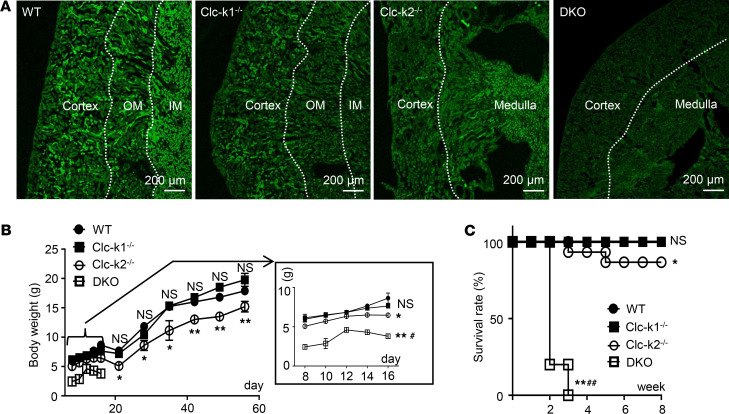
Phenotypes of Clc-k1^–/–^, Clc-k2^–/–^, and Clc-k1^–/–^ Clc-k2^–/–^ DKO mice. (**A**) IFC staining of Clc-k channels in longitudinal sections of kidneys from 8-week-old WT and Clc-k–knockout mice (except DKO mice, which cannot survive to adulthood, for which 2-week-old kidney was used). White dotted lines mark the boundaries between the cortex, outer medulla (OM), and inner medulla (IM). Clc-k2^–/–^ and DKO kidneys did not have a clear separation between the OM and IM. Scale bars: 200 μm. (**B**) Body weight of WT and Clc-k–knockout mice from day 8 to 8 weeks. Inset: Magnified view of data for days 8–16 (*n* = 12 for each group). Results are presented as mean ± SEM, and data were analyzed by 2-way ANOVA with mixed-effects analysis. (**C**) Survival of WT and Clc-k–knockout mice (*n* ≥ 12 for each group). Data were analyzed by log-rank test. *P < 0.05, **P < 0.001, and NS between designated group and WT mice; ^#^*P* < 0.05, ^##^*P* < 0.001 between Clc-k2^–/–^ and DKO mice.

We generated mice with Ksp-Cre–mediated constitutive kidney-specific deletion of Clc-k1 (Clc-k1^–/–^), Clc-k2 (Clc-k2^–/–^), or both (DKO) in the same C57BL/6JNarl background (Supplemental Figure 3, A–D). The currently available anti–Clc-k antibody detected both Clc-k1 and Clc-k2. The molecular sizes of Clc-k1 and Clc-k2 are similar, precluding their separation by Western blotting (Supplemental Figure 4). We hence analyzed protein expression of Clc-k in WT and gene-deleted mice using IFC. As shown in Figure 4A, IFC staining by the pan–Clc-k antibody revealed signals in the cortex and medulla in WT mice but no signals in DKO mice. Signal was limited to tubules within cortex and outer medulla in Clc-k1–deleted and to outer and inner medulla in Clc-k2–deleted kidneys. These results validated the gene deletion and supported the finding revealing differential expression of Clc-k1 and Clc-k2 in the mRNA studies.

Pregnant females carrying Clc-k2–null or DKO pups had polyhydramnios and gave birth to fewer offspring than WT females, but pups were born at the expected Mendelian ratios (data not shown). After birth, Clc-k2–null and DKO pups were lethargic and growth retarded compared with WT and Clc-k1–null littermates (Figure 4B and Supplemental Figure 3E). After birth, DKO pups failed to gain body weight, and all died by 3 weeks (Figure 4B, inset, and Figure 4C), and neither saline nor indomethacin injection extended life (*n* > 12 for each group, data not shown). Clc-k2–null pups had better weight gain than DKO mice; nevertheless, approximately 15% of them died by 5 weeks of age.

Because DKO mice did not survive beyond 3 weeks, the phenotypes of 2-week-old age-matched mice were analyzed (Table 1). Compared with WT littermates, Clc-k1–null mice had lower urinary osmolality and higher urinary sodium excretion, whereas Clc-k2–null mice excreted more sodium, potassium, and divalent cations, leading to lower serum levels of sodium, potassium, chloride, and calcium. The severity of volume and electrolyte loss in Clc-k2–null relative to Clc-k1–null mice was evidenced by metabolic alkalosis and elevation in blood urea nitrogen (BUN) in the former. Compared with Clc-k2–null mice, DKO mice excreted even more sodium, potassium, and divalent cations, and developed severe hypovolemia and acute kidney injury. Overall, Clc-k1–null mice surviving to adulthood excreted approximately 4-fold more urine and slightly more divalent cations than WT mice, without an increase in total sodium excretion. Adult Clc-k2–null mice developed chronic kidney disease with high serum urea and creatinine levels in addition to the electrolyte disorder (Table 2).

**Figure 5:**
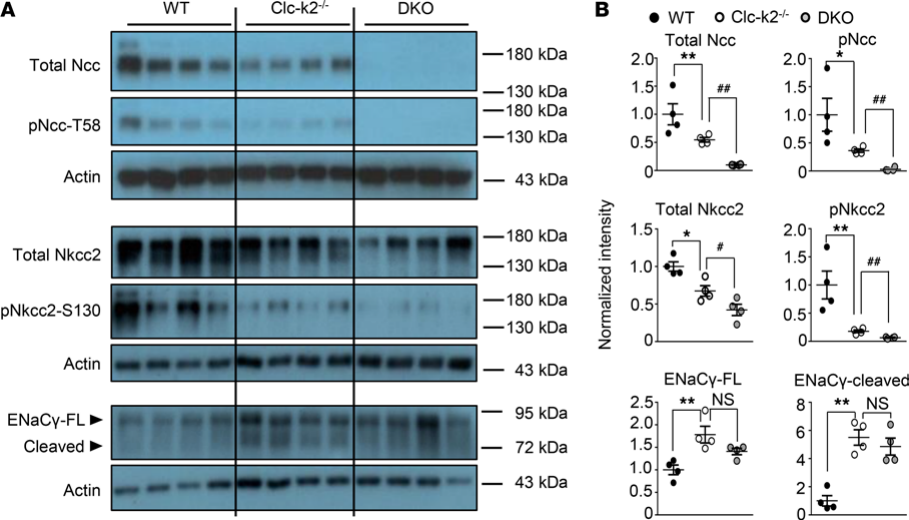
Comparison of Ncc, Nkcc2, and ENaC protein abundance in 2-week-old WT, Clc-k2^–/–^, and Clc-k1/k2 DKO kidneys. (**A**) Representative WBs of 4 experiments with similar results. (**B**) Quantitative measurements of each protein’s expression, which was normalized to the amount of β-actin and reported relative to WT controls. The abundance of each band was measured by densitometry by the ImageJ program. Bonferroni’s correction was used for multiple comparisons. *P < 0.05, **P < 0.001 between Clc-k2^–/–^ and WT mice; ^#^*P* < 0.05, ^##^*P* < 0.001, and NS between Clc-k2^–/–^ and DKO mice.

Renal tubules and transporters undergo adaptation. To assess the impact of salt wasting from Clc-k deletions on other transporters, we quantified expression of transporters as well as their functional activity using diuretic sensitivity analysis. Due to the shortened life span of DKO mice, we studied 2-week-old DKO mice. We relied on levels of phosphorylated transporters as an index of activity due to technical difficulties of administering diuretics and timed collection of urine in these mice. In 2-week-old kidneys, the level of total and phosphorylated Ncc and Nkcc2 protein was reduced in DKO and Clc-k2–null versus WT mice, with a much greater reduction in DKO than in Clc-k2–null mice (Figure 5). Epithelial sodium channel (ENaC) activity reflected by cleavage of γ-subunit increased in DKO and Clc-k2–null mice. Overall, these results support the notion that Clc-k2 is highly expressed in TAL and DCT and that loss of Clc-k results in downregulation (i.e., disuse atrophy) of transporters in these segments. Clc-k channels are much less abundant in CCD and probably present in non-ENaC-containing intercalated cells (19), such that their loss results in compensatory upregulation of ENaC downstream.

**Figure 6:**
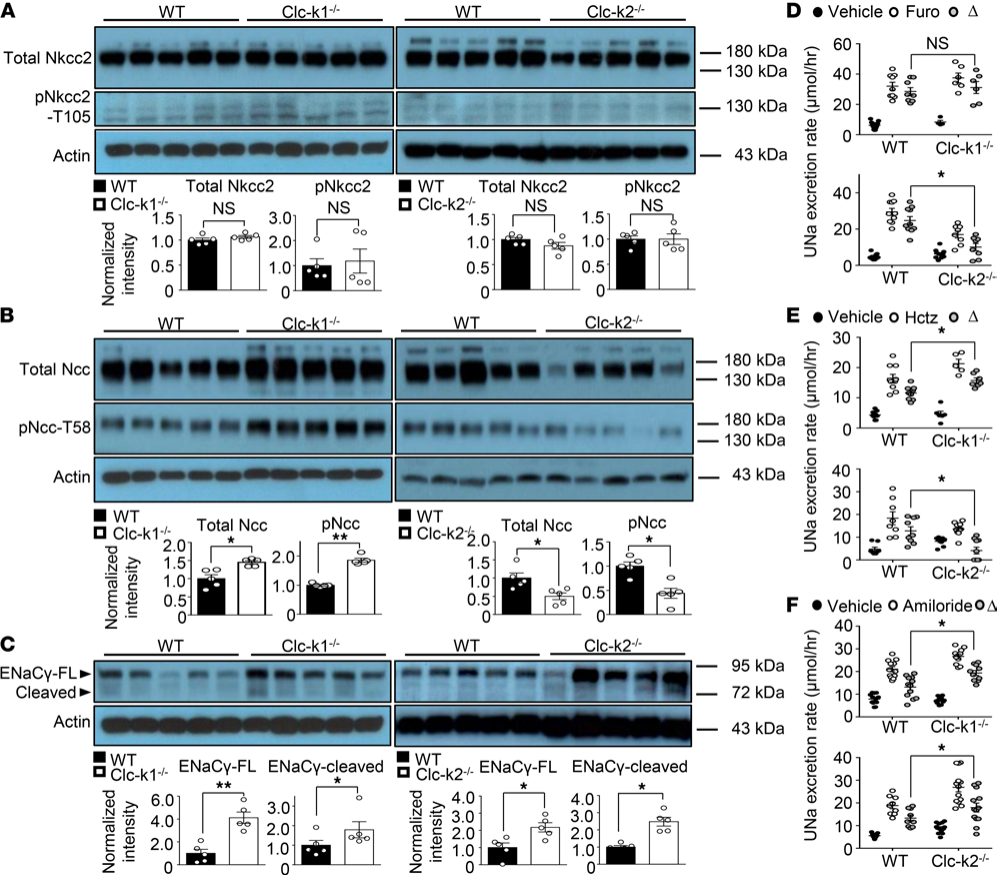
Protein abundance and activity of Ncc, Nkcc2, and ENaC in 8-week-old Clc-k1–null and Clc-k2–null mice. (**A–C**) WB analyses of Nkcc2 (**A**), Ncc (**B**), and ENaC (**C**) in 8-week-old WT, Clc-k1^–/–^, and Clc-k2^–/–^ mice. Actin loading controls are shown for each blot. Protein expression was normalized to the amount of β-actin and reported relative to WT controls. The abundance of each band was measured by densitometry by the ImageJ program. The results shown here are representative of 5 experiments with similar results. *P < 0.05, **P < 0.001, and NS between the indicated groups using unpaired *t* test. (**D–F**) Diuretic challenge tests reflect the in vivo activity of Nkcc2 (**D**), Ncc (**E**), and ENaC (**F**) in 8-week-old WT, Clc-k1–null, and Clc-k2–null mice. The effects of furosemide (Furo) (15 mg/kg), hydrochlorothiazide (Hctz) (12.5 mg/kg), or amiloride (0.65 mg/kg) versus vehicle (Veh) on urinary Na^+^ (Una) excretion rate were calculated. Diuretic-sensitive natriuresis (Δ) in WT, Clc-k1^–/–^, and Clc-k2^–/–^ mice (*n* = 6 for each group) were compared. Results are presented as mean ± SEM. Mice in the same experiment were compared. *P < 0.05 and NS between indicated groups using unpaired *t* test.

We assessed the effect of the loss of Clc-k1 in 8-week-old adult kidneys. Clc-k1–null versus WT mice had normal Nkcc2 activity, as reflected by comparable levels of phosphorylated Nkcc2 protein and furosemide-sensitive Na^+^ excretion (Figure 6, A and D). The activity of Ncc and ENaC in 8-week-old Clc-k1 mice was increased versus that in WT mice, as reflected by higher levels of phosphorylated Ncc and thiazide-sensitive Na^+^ excretion (Figure 6, B and E) and by higher levels of the cleaved form of γENaC and amiloride-sensitive Na^+^ excretion (Figure 6, C and F), respectively. These findings that Clc-k1–null mice have unchanged Nkcc2 activity but upregulated Ncc and ENaC activity suggest that the role of Clc-k1 in NaCl reabsorption is predominant in TAL. This is consistent with gene expression results indicating that Clc-k1 is restricted to tAL and mTAL (Figure 3). The reason for the apparent lack of change in Nkcc2 activity in Clc-k1–null mice may be compensatory upregulation of Clc-k2. Indeed, we found that the Clc-k2 transcript was upregulated in 2-week-old and 8-week-old Clc-k1–null mice (Supplemental Figure 5). Conversely, the Clc-k1 transcript was upregulated in 2-week-old Clc-k2–null mice, further supporting that Clc-k1 and Clc-k2 work together and compensate for each other during the neonatal period. Clc-k1 transcript, however, was reduced in 8-week-old Clc-k2–null mice, probably due to medulla hypoplasia at this stage (see below). This finding, along with the differential localization, suggests that the function of Clc-k1 cannot compensate for Clc-k2 deficiency in adult mice.

The larger size of 8-week-old mice allows assessment of the functional activity of transporters by diuretic sensitivity (Figure 6, D–F), as well as phosphorylated protein abundance (Figure 6, A–C). We, therefore, studied 8-week-old Clc-k2–null mice for comparison with Clc-k1–null mice. The results demonstrated that, relative to WT, functional activity of Nkcc2 and Ncc was decreased in Clc-k2 mice, while ENaC activity was increased. These results in 8-week-old Clc-k2–null mice were qualitatively similar to those in 2-week-old mice, as shown in Figure 5. Together with the gene expression data (Figure 3), IFC staining (Figure 4), and whole animal clearance data (Tables 1 and 2), the results provide support for the notion that Clc-k1 contributes to renal NaCl reabsorption and the predominant site of action is in TAL.

**Figure 7:**
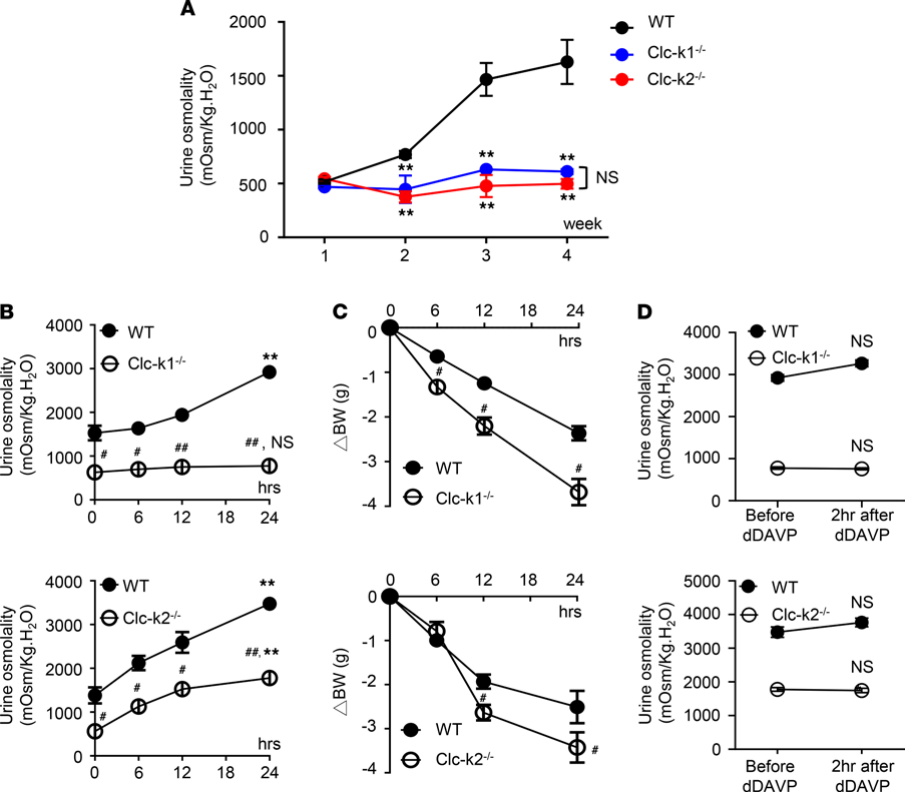
Water deprivation and DDAVP test in Clc-k1–null and Clc-k2–null mice. (**A**) Urine osmolality for WT, Clc-k1^–/–^, and Clc-k2^–/–^ mice (*n* = 6 for each group) during postnatal weeks 1–4 with free access to drinking water. Results are presented as mean ± SEM. **P < 0.001 between the designated group and WT mice; and NS between Clc-k1^–/–^ and Clc-k2^–/–^ mice using 2-way ANOVA with mixed-effect analysis. (**B** and **C**) 8-week-old Clc-k1–null, Clc-k2–null, and WT mice received water deprivation and DDAVP test, with serial measurements of urine osmolality (**B**) and BW change (**C**) during water deprivation. **P < 0.001, NS between the 0-hour and 24-hour time points; ^#^*P* < 0.05, ^##^*P* < 0.001 between the designated group and WT mice using 2-way ANOVA with mixed-effect analysis. (**D**) Urine osmolality before and after intraperitoneal DDAVP injection (*n* = 6 for each group). NS between baseline and 2 hours after DDAVP (0.4 ng/g body weight) injection by 1-way repeated-measure ANOVA.

Urinary concentration increased over the first 4 weeks of life in WT mice. In contrast, urine osmolality in Clc-k1–null and Clc-k2–null mice was low and did not increase with age (Figure 7A). In adulthood, Clc-k1–null mice continued to excrete dilute urine during water deprivation, while Clc-k2–null mice excreted suboptimally concentrated urine despite similar urine output in the basal condition (Figure 7, B and C). Clc-k1–null mice failed to respond to an injection of 1-deamino-8-d-arginine vasopressin (DDAVP) after adequate water deprivation (Figure 7D), indicating complete nephrogenic diabetes insipidus (NDI). Clc-k2–null mice partially responded to water deprivation and DDAVP, indicating partial NDI.

### Effects of Clc-k2 deficiency on the structure of neonatal kidneys.

**Figure 8:**
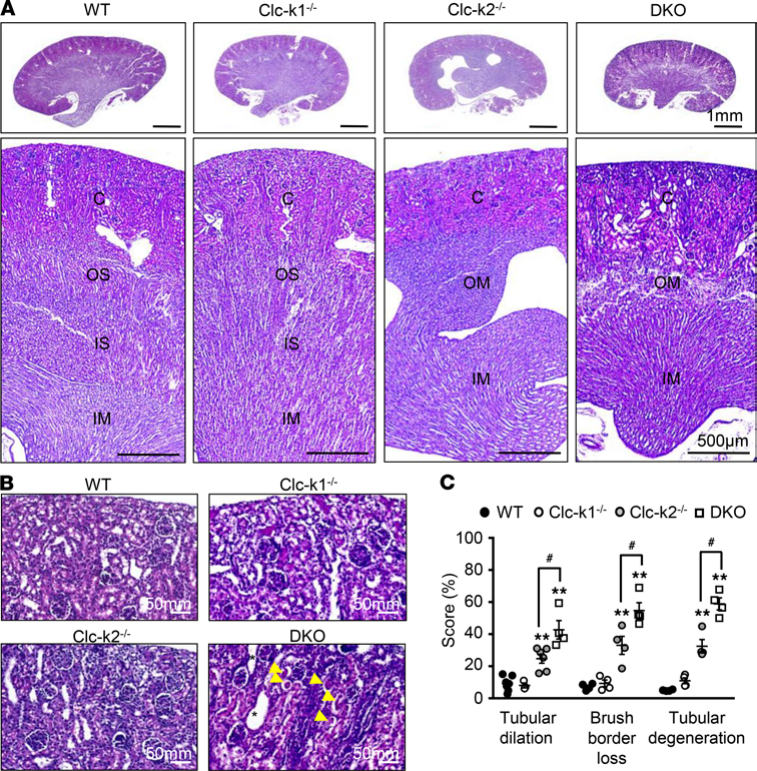
Effects of Clc-k deficiency on renal histology. (**A**) Top row: H&E-stained kidney longitudinal sections of 2-week-old WT, Clc-k1^–/–^, Clc-k2^–/–^, and DKO kidneys (scale bar: 1 mm). Bottom row: Approximate renal areas (scale bars: 500 μm). C, cortex, OS, outer stripe of the outer medulla; IS, inner stripe of the outer medulla; OM, outer medulla; IM, inner medulla. (**B**) H&E-stained renal cortex of WT, Clc-k1^–/–^, Clc-k2^–/–^, and DKO kidneys. In DKO kidneys, the dilated tubules are marked by asterisks, and yellow arrowheads label tubules with desquamation and brush border loss. Both Clc-k2^–/–^ and DKO kidneys are characterized by marked mononuclear cell infiltration in the interstitium and tubular cells with prominent nucleoli, especially those in medullary rays. Scale bars: 50 mm. (**C**) Quantitation of the degree of tubular injury (32). **P < 0.001 between the designated group and WT mice; ^#^*P* < 0.05 between Clc-k2^–/–^ and DKO mice using 1-way ANOVA with Šidák’s test for multiple comparisons.

The 2-week-old Clc-k–null kidneys were examined under light microscopy. Compared with those of WT and Clc-k1–null littermates, Clc-k2–null and DKO kidneys showed a poorly developed outer medulla without a clear separation between the inner and outer stripes (Figure 8A). In the renal cortex, Clc-k2–null kidneys showed enlarged Bowman’s space and dilated renal tubules. DKO kidneys displayed severe tubular injury, with desquamation, luminal dilatation, loss of brush border, epithelial cells with prominent nucleoli and scant cytoplasm, and mononuclear cell infiltration (Figure 8, B and C). These changes were much more notable in tubules in the medullary rays, including the S3 segment of the proximal tubule and TALs.

**Figure 9:**
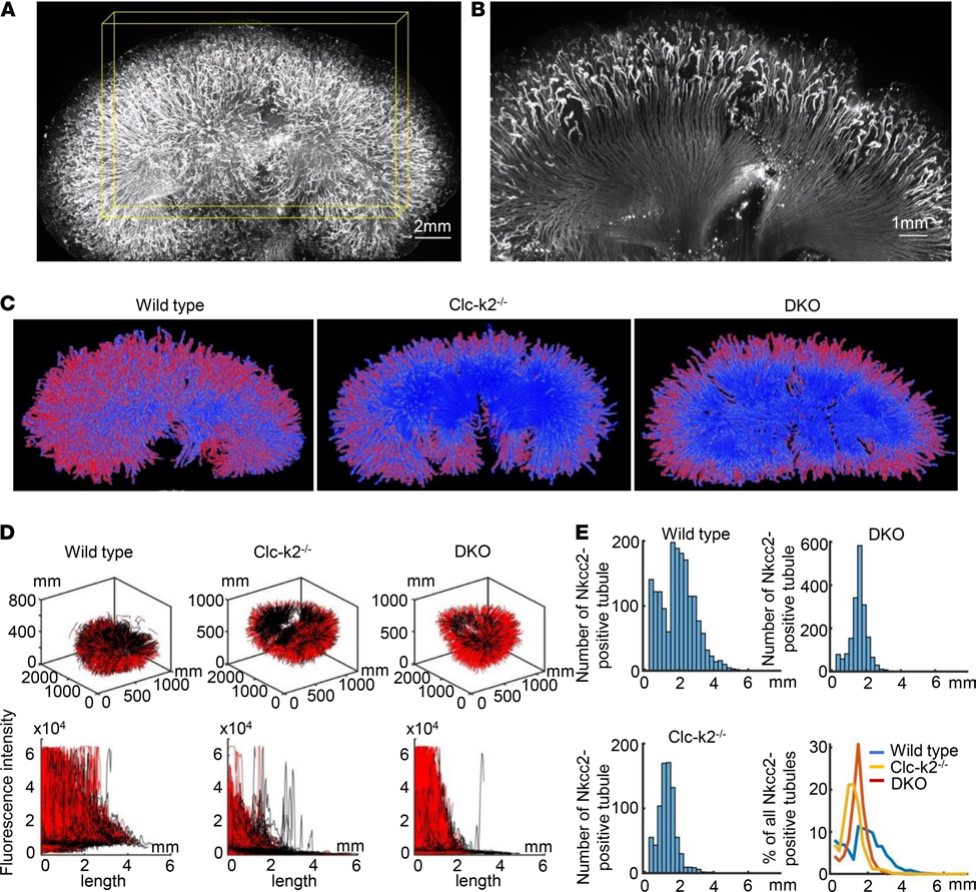
3D imaging of whole expanded mouse kidneys. (**A**) Whole-kidney view of Nkcc2-positive TALs. Scale bar: 1 mm. (**B**) Magnified view of a cross-section (marked by a yellow rectangle in **A** of Nkcc2-positive TALs. Scale bar: 1 mm. (**C**) 3D reconstruction of Nkcc2-positive TALs based on the fluorescence intensity of Nkcc2 (red: strong, blue: weak). (**D**) Quantitative Nkcc2 fluorescence analysis of whole-kidney TALs. The tracked renal tubules (black line) and NKCC-positive signal (red line) were plotted together in 3D space (upper panel) and as intensity versus length (lower panel). Fluorescence intensity of each TAL was linearized from the cortical end (high intensity) to the medullary end (low intensity). The *x* axis indicates the length of expanded TALs (not the real length in vivo). (**E**) Length analysis of whole-kidney TALs, ranging from 0.5 mm to 6 mm, in 250 μm increments. Note that the sample is physically expanded to approximately 4 times larger in each dimension.

To further characterize TAL development, we measured TAL length in optically cleared 1-week-old Clc-k–null kidneys that were expanded for high-resolution 3D imaging (Supplemental Figure 6). The whole-kidney Nkcc2-positive TALs were visualized using scanning Bessel beam light-sheet fluorescence microscopy (Figure 9, A and B, and Supplemental Video 1). The Nkcc2 fluorescence signal of each TAL was tracked with the Cylinder Correlation module in Amira and reconstructed into a 3D whole-kidney view (Figure 9C). The Nkcc2 fluorescence intensity along each tracked TAL was analyzed (Figure 9D). Compared with those in the WT kidney, the Nkcc2-positive TALs in Clc-k2–null and DKO kidneys were disproportionately short (Figure 9E), providing direct visual and quantitative confirmation for developmental defects of TAL caused by Clc-k2 deficiency.

### Developmental defect of Clc-k2 deficiency in the medulla is vital for the salt-wasting phenotype.

**Figure 10:**
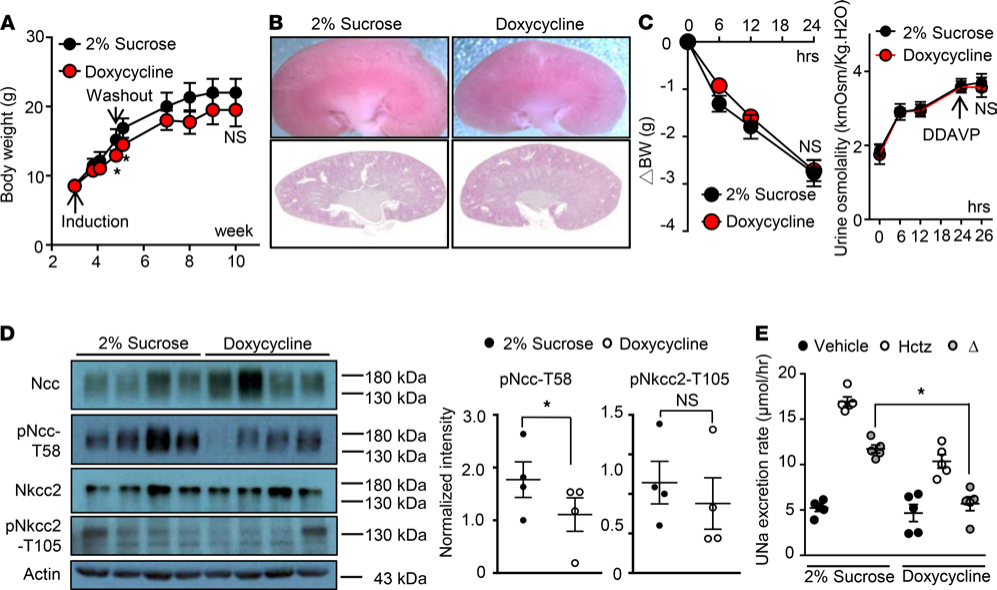
Postneonatal Clc-k2 deletion causes a milder phenotype. (**A**) BW from the beginning of induction (week 3) to week 10. Results are presented as mean ± SEM, and data were analyzed by 2-way ANOVA with mixed-effects analysis. Doxycycline’s side effects likely cause the difference at weeks 5 and 6. (**B**) Gross appearance and H&E staining of the longitudinal section of a doxycycline-induced Clc-k2 deficient kidney (Doxycycline) and a control kidney (2% Sucrose). (**C**) Water deprivation and DDAVP tests were performed on 10-week-old doxycycline-induced Clc-k2–deficient mice and controls. NS between Clc-k2–deficient and control groups and between baseline and 2 hours after DDAVP (0.4 ng/g body weight) injection by 2-way ANOVA with mixed-effect analysis. (**D**) WB analysis of total and phosphorylated levels of Ncc and Nkcc2 in 10-week-old inducible Clc-k2–deficient kidneys and controls. Actin loading controls are shown for each blot. Protein expression was normalized to the amount of β-actin and reported relative to controls. The abundance of each band was measured by densitometry by the ImageJ program. Results shown are representative of 4 experiments with similar results. Results are presented as mean ± SEM. Mice in the same experiment were compared. *P < 0.05 and NS between Clc-k2–deficient mice (Doxycycline) and controls (2% Sucrose) using unpaired *t* test. (**E**) Hctz (12.5 mg/kg) challenge test reflects the in vivo activity of Ncc in 10-week-old inducible Clc-k2–deficient mice and controls. *P < 0.05 between Clc-k2 deficient mice and controls using unpaired *t* test.

To determine the impact of Clc-k2 deficiency during neonatal kidney development, we generated mice carrying a floxed allele of the *Clcnk2* gene and the doxycycline-inducible kidney-specific Cre driver Pax8/LC1 (Pax8/LC1/Clc-k2^fl/fl^) (Supplemental Figure 7). We induced Clc-k2 deletion at 2 time points: beginning at 3 weeks of age, when the maturation of medulla is complete; and during the embryonic stage, by feeding pregnant females before and during pregnancy. At 10 weeks, the phenotype of embryo-induced Clc-k2–knockout mice was similar to the presentation of constitutive Clc-k2–null mice (Table 3 versus Table 2). In contrast, mice with postnatally induced Clc-k2 knockout had normal weight gain and gross appearance of the renal medulla as well as intact urine-concentrating ability as compared with controls (Figure 10, A–C). Blood and urine analysis in mice with conditional postneonatal Clc-k2 deletion versus non-induced control mice revealed slightly increased urinary potassium excretion, hypokalemia, normocalciuria, and mild hypovolemia evidenced by a slight elevation in BUN and plasma renin activity, and metabolic alkalosis. Relative to those in constitutive or embryo-induced Clc-k2–null mice, these abnormalities were milder. Analysis of transporters revealed that Ncc activity was decreased, while Nkcc2 activity was unchanged (Figure 10, D and E), indicating that defects resided in the cortex (DCT), not in the medulla (mTAL). Overall, it is unlikely that these differences in phenotypes between postneonatal and embryonic deletion of Clc-k2 were caused by different efficiencies in gene deletion, as the reduction in Clcnk2 transcript was approximately 95% in each line, although we cannot totally exclude this possibility (Supplemental Figure 8). The results support the notion that Clc-k2 activity is required for perinatal and neonatal medulla development. Salt wasting in constitutive Clc-k2–deficient mice was in large part due to defects in medulla development. In medulla-intact Clc-K2–deficient mice, salt wasting was predominantly the result of reduced Ncc activity in the DCT.

## Discussion

Our current study focusing on developing and neonatal kidney reveals several insights into the roles of Clc-k channels. We found that Clc-k1 and Clc-k2 were diffusely expressed in the late embryonic kidney and overlapped substantially, rather than being differentially localized to the inner medulla and outer medulla/cortex, as currently believed for the adult kidney. During the first 2 weeks after birth, Clc-k1/k2 expression became differentially localized, which coincides with the maturation of the loop of Henle and renal medulla. By constitutive deletion of Clc-k channels, we found that Clc-k2 plays a major role in renal NaCl reabsorption. Clc-k1 still contributed to salt reabsorption: double deletion of Clc-k1 and Clc-k2 caused severe salt wasting, leading to animal death in 3 weeks. Beginning at around 1 week of age, Clc-k1 was gradually confined to the inner medulla, where it primarily serves to control urine concentration in the thin limb. In addition to TAL, both channels likely also underlie the basolateral chloride conductance of the neonatal DCT, because DKO mice had greater loss of Ncc protein and inevitable neonatal fatality not amenable to salt repletion. The notion that Clc-k1 and Clc-k2 function together in TAL and DCT up to the perinatal period likely explains why patients with classic Bartter syndrome with ClC-Kb mutation tend to have less-severe perinatal salt wasting than those with antenatal Bartter with NKCC2 or ROMK mutations.

Our studies also reveal unexpected roles of Clc-k channels in the development of the renal tubule and medulla. Clc-k2–null mice had a poorly developed medulla. Besides evidence of medulla hypoplasia, advanced 3D imaging of optically cleared kidneys revealed that TAL was shortened in these mice. Importantly, the requirement of Clc-k2 in perinatal medulla development has significant functional consequences. Mice with *Clcnk2* gene conditionally deleted after medulla maturation was complete did not exhibit medulla hypoplasia and had a milder phenotype than mice with constitutive or embryo-induced *Clcnk2* deletion. The medulla-intact Clc-k2–deficient mice had intact Nkcc2 activity and decreasing Ncc activity, further supporting the functional integrity in the medulla. Patients with Bartter syndrome have germline mutations of ClC-Kb, raising interesting questions regarding renal medulla hypoplasia in Bartter patients. Renal biopsy is rarely, if ever, performed in Bartter infants. However, renal ultrasound findings in patients with Bartter syndrome include loss of corticomedullary differentiation, hyperechoic renal medullary pyramids, and no identifiable renal pyramids, suggesting defects in medulla development (20, 21).

How loss of Clc-k2 (or ClC-Kb) function impairs renal medulla and TAL development is currently unknown. Mice with constitutive Clc-k2 deletion manifested polyuria and partial NDI, as observed in some patients with classic Bartter syndrome (22). The structural changes in the medulla cannot be simply explained by polyuria-induced hydronephrosis, since Clc-k1–null mice show a normal renal medulla structure despite a similar degree of polyuria. Use hypertrophy and disuse atrophy are common adaptive changes in renal tubules. Maturation and development of renal medulla occur in the neonatal period of mouse kidneys. The function of Clc-k2 in mTAL during that period may conceivably play a role in medulla development. Alternatively, the cell-autonomous effect of Clc-k2 channel through regulation of intracellular chloride concentration and/or membrane potentials or hypovolemia-mediated tubular injury (see below) may be the mechanisms.

The renal histology of Clc-k2–null and DKO neonatal kidneys is consistent with ischemic tubular injury from volume depletion. Patients with ClC-Kb mutations have variable disease onset ranging from perinatal to adolescent. Our current findings that Clc-k1 also contributed to NaCl reabsorption in the TAL and DCT and that hypovolemia induces tubular injury in the perinatal kidney offer potential explanations for phenotypical variability in patients with ClC-Kb mutations. In this scenario, volume loss when it reaches a critical threshold may induce a feed-forward vicious cycle to amplify disease. ClC-Kb–deficient individuals, being less likely to experience large volume loss at birth, may have variable presentation depending on volume status and whether the volume loss crosses the threshold. Regarding the potential relevance of the findings in mice to the human kidney, it is worth noting that while human nephrogenesis (formation of new nephrons) is more advanced than in mice and completes before birth, human kidneys, like mouse kidneys, continue to grow at a rapid rate after birth (23). Unlike nephrogenesis, human renal medulla development and functional maturation constitute a primarily ex utero event that takes 12–18 months after birth to complete, compared with 3–4 weeks in mice (24, 25).

In contrast to previous reports on Clc-k2–null mice (15, 17), our Clc-k2–null mice uniformly showed impaired renal function in adulthood, a prevalence much greater than that of chronic kidney disease (CKD; ~25%) in patients with classic Bartter syndrome (7). This may be due to genetic background or the fact that our mice received no treatments. Hypovolemia-induced ischemic tubular injury leads to progression to CKD (26, 27). Our histological findings of tubular injury in Clc-k2–null kidneys support the notion.

**Figure 11:**
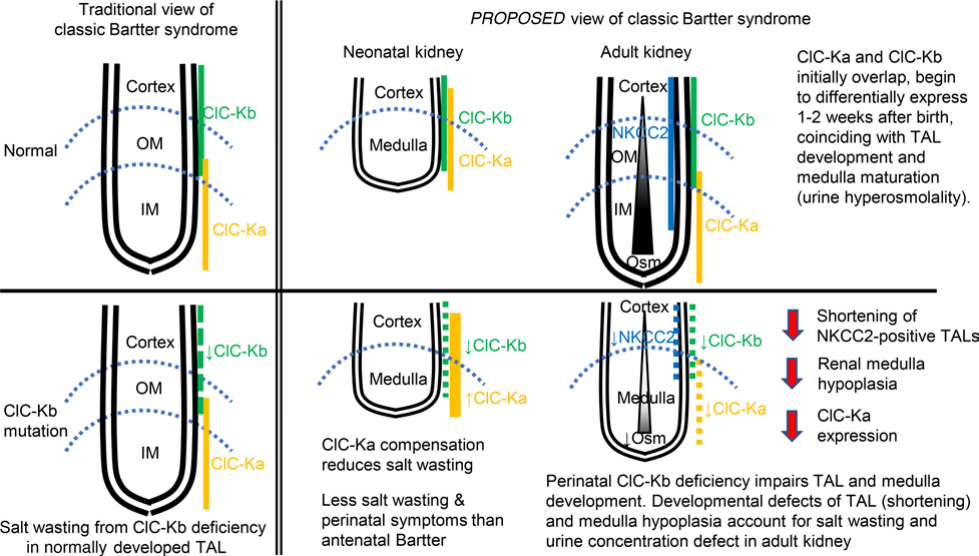
Proposed pathogenesis of classic Bartter syndrome. Left: traditional view; right: novel view. Top: normal physiology; bottom: classic Bartter syndrome with ClC-Kb mutation.

In conclusion, by investigation of mice with and without Clc-k1 and Clc-k2 during development and in postnatal life, we provide insights into the functions of these channels and the disease pathogenesis of classic Bartter syndrome (Figure 11). As opposed to differential localization to the inner medulla and outer medulla/cortex in adult kidneys, Clc-k1 and Clc-k2 were broadly expressed and colocalized in embryonic and early neonatal kidneys. Dual roles of Clc-k1 and Clc-k2 in neonates likely explain the less-severe phenotypes of patients with classic Bartter syndrome. Importantly, loss of function of Clc-k channels in the early stage impaired medulla and TAL maturation, which was the main cause of salt wasting in adulthood. Future studies will decipher the precise mechanisms underlying how Clc-k deficiency affects renal tubule development.

## Methods

### In situ hybridization of Clcnk1 and Clcnk2 mRNA in kidneys.

In situ RNA hybridization was performed using RNAscope 2.5 HD–BROWN assay (Advanced Cell Diagnostics [ACD], 322300) according to the manufacturer’s protocol. Briefly, kidneys harvested from mice of ages ranging from E13 to 8 weeks were fixed in formalin, embedded in paraffin, and cut into sections of 5–20 μm. The kidney sections were deparaffinized in xylene and 100% ethanol, air dried for 5 minutes, and covered by 3% hydrogen peroxide for 10 minutes. After target retrieval for 15 minutes at 95°C, the kidney slides were digested by Protease Plus for 30 minutes at 40°C and hybridized with Clc-k1 or Clc-k2 probes for 2 hours at 40°C. The slides were then washed by a 1× wash buffer following each amplification step. The RNA signals were revealed by incubating slides with BROWN-A and -B solution for 10–60 minutes, counterstaining with 12.5% hematoxylin, and mounting with Sub-X medium (Leica Biosystems, 3801740). The following RNAscope probes were custom designed by ACD: Clcnka (NM_001146307.1, region 2-2389; 536031), Clcnkb (NM_019701.2, region 591-2341; 528541).

### Generation of Clc-k–knockout mouse models and balance studies.

All mouse models in this study were newly generated by us and in the same C57BL/6JNarl background (see Supplemental Methods). Mice with floxed alleles of *Clcnk1*, *Clcnk2*, and both *Clcnk1* and *Clcnk2* were generated using CRISPR/Cas9. It should be noted that the mouse *Clcnk1* and *Clcnk2* genes are closely adjacent to each other in the same chromosome (chromosome 4) separated by approximately 5.6 kb. Mice homozygous for floxed alleles were crossed with Ksp-Cre transgenic mice (purchased from the National Laboratory Animal Center, Taipei, Taiwan) (28) to produce constitutive kidney-specific Clc-k1–null (Clc-k1^–/–^), Clc-k2–null (Clc-k2^–/–^), and DKO mice (see Supplemental Methods). The Pax8-rtTA/LC1 system (a gift from Shuei-Liong Lin, National Taiwan University) was used to generate an inducible kidney-specific Clc-k–knockout mouse model (29). Three-week-old Pax8-rtTA/LC1/Clc-k2^fl/fl^ mice were given 2% sucrose drinking water containing 2 mg/mL doxycycline for 2 weeks to induce recombination at floxed sites, while the control littermates were given 2% sucrose drinking water. Experiments were initiated after a 2-week washout of doxycycline. To induce embryonic Clc-k2 knockout, Pax8-rtTA/LC1/Clc-k2^fl/+^ female mice were given 2% sucrose drinking water containing 2 mg/mL doxycycline before and during pregnancy. To rescue DKO mice, subcutaneous saline (60 μL/g body weight) or indomethacin (6 μg/g body weight, MilliporeSigma) was injected beginning at birth. Blood samples were collected via cardiac puncture. Spot urine samples were collected by applying pressure to the bladder of 2-week-old neonates; for 8-to 10-week-old adult mice, 24-hour urine samples were collected by the metabolic cage. Plasma biochemisty was measured and gas analyzed by iSTAT (Abbott), while plasma renin activity was determined by a Mouse Renin 1 ELISA Kit (Thermo Fisher Scientific). Urine biochemistry was measured by an automated chemistry analyzer (Olympusm AU 5800) and normalized by body weight (for 24-hour urine samples) and/or urine creatinine (for spot urine samples). Systolic and diastolic blood pressures were measured using the tail-cuff method, and diuretic tests were performed as previously described (30). For water deprivation, water was withheld from the mice for 24 hours, and urine samples were collected at 0, 6, 12, 24 hours. DDAVP (0.4 ng/g body weight, MilliporeSigma) was injected intraperitoneally after water deprivation, followed by an additional urine sample collection 2 hours later.

### Western blot analysis, renal histology, and immunohistochemical and IFC staining.

Kidneys were harvested from mice immediately after sacrifice for Western blot (WB), immunohistochemical, and IF analyses as previously described (30). Primary antibodies were applied overnight at 4°C, followed by secondary antibodies for 2 hours at room temperature. The primary antibodies used in this study included anti–ClC-K antibody (1:2000 dilution for WB; 1: 200 dilution for IF) (6), anti-NCC antibody (1:5000 dilution for WB; 1:200 dilution for IF; Millipore, AB3553), anti-NCC phospho-Thr58 antibody (1:2000 dilution for WB) (30), anti-NKCC2 (1:2000 dilution for WB; 1:200 dilution for IF; Millipore, AB2281), anti-NKCC2 phospho-Ser130 (1:1000 dilution for WB; Dundee, S432C), anti-NKCC2 phospho-Thr105 (1:1000 dilution for WB; Dundee, S378C), anti-γENaC antibody (1:1000 dilution for WB; StressMarq Biosciences, SPC-405), and anti-Barttin (1:50 dilution for IHC, Santa Cruz Biotechnology Inc., sc-365161; another one provided by Shinichi Uchida, Tokyo Medical and Dental University) (31). The secondary antibodies used in this study included anti-rabbit (1:4000 dilution; The Jackson Laboratory, 111-035-003) and anti-sheep IgG antibodies (1:10,000 dilution; Bethyl, A130-101P). IFC images were obtained by confocal microscopy (Zeiss LSM 880), while IHC and H&E-stained samples were scanned by Zeiss Axioscan Z1. Tubulointerstitial injury was defined as tubular damage, atrophy, interstitial inflammation, and fibrosis, and scored as previously described (32).

### Microdissection and reverse transcription PCR.

C57BL/6JNarl mice at the age of 6–8 weeks were used for microdissection. Slices of kidney were placed into a prewarmed collagenase type I (Worthington) (1.5 mg/ml dissolved in DMEM/F12) solution in a 15 mL tube, which was then shaken vigorously on a titer plate shaker at 37°C for 5–15 minutes. After digestion, the individual nephron segments were dissected in 4°C Hank’s solution and collected by adhering the tubules to small glass beads (0.5mm diameter; Thomas Scientific) and then transferring the beads to 1.5 mL tubes containing 0.6 mL RNase inhibitor–containing lysis buffer. Total RNA was immediately extracted using a Quick-RNA MicroPrep kit (Zymo Research). Reverse transcription was performed using an Applied Biosystems High-Capacity cDNA Reverse Transcription Kit (Thermo Fisher Scientific). Quantitative real-time PCR (RT-PCR) was carried out on a LightCycler 480 System (Roche). We verified that no amplification was produced when reverse transcription was omitted from the sample. Sequences of primers for RT-PCR analysis were as follows: Clc-k1, GTACCACCCACGGTTCACCA (forward) and AGACAGGGCCTCTCCCAAGA (reverse); Clc-k2, CCAAGGTGGTGGGCCTCTC (forward) and GCCCAGGTAAGCAGCGATCA (reverse); Nkcc2, CCAGAGCGTTGTCTAAAGCA (forward) and TGGGCAGCTGTCATCACTTA (reverse); Ncc, GGGTTTGTGTCATGAGGATG (forward) and CTCGTCCGATCGTGGTAGA (reverse); Barttin, GGGTACATTCCTTATCAGCC (forward) and TTGGAAGTCAGAGTCTGCTG (reverse); Aqp2, CTGGCTGTCAATGCTCTCCAC (forward) and TTGTCACTGCGGCGCTCATC (reverse); and Gapdh, CGTCCCGTAGACAAAATGGT (forward) and TCAATGAAGGGGTCGTTGAT (reverse). Relative mRNA abundance of genes of interest was standardized to the abundance of *Gapdh* mRNA and calculated by ΔΔCt value (2*Ct value of GAPDH – Ct value gene of interest*). For comparison between Clc-k1 and Clc-k2, the efficiency of each primer set in RT-PCR assay was calculated from the results of serial (0-, 10-, 100-fold) dilutions of cDNA, which encompass our detecting range (33). The slope of threshold cycles obtained from Clc-k1 and Clc-k2 reactions of serially diluted sample tubular cDNA was used to calculate the corresponding efficiencies (*E*) according to the folowing equation: 
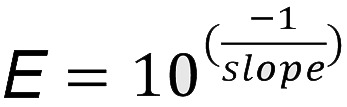
 To analyze the relative abundance of Clc-k1 and Clc-k2, the mRNA level of Clc-k2 in each whole-kidney lysate sample was standardized to Clc-k1 (defined as 1). Each sample was assayed in triplicate.

### Tissue expansion of kidneys and 3D data acquisition with scanning Bessel beam light-sheet microscopy.

Seven-day-old WT, Clc-k2–null, and DKO kidneys were optically cleared and expanded before incubation in anti-Nkcc2 antibody (AB2281). The harvested and perfused 7-dayold kidneys were preincubated in gelling solution overnight at 4°C (34) and the solution was replaced with fresh gelling solution on the second day, followed by incubation at 37°C for 2 hours in a humidified gelation chamber. After the gel polymerization, excess gel around the samples was removed, and the samples were denatured with SDS at 70°C with gentle shaking for 2 days (35). Immunostaining was performed after the SDS denaturation and thorough washout of the SDS. Samples were blocked first with 5% NGS, 0.4% Triton X-100 in 1× PBS buffer overnight at 37°C; then incubated in primary antibody (1:50 dilution of rabbit anti-Nkcc2; AB2281) in blocking buffer for 3 days, and subsequently in secondary antibody (1:50 dilution of donkey anti–rabbit-488; Invitrogen, A21206) in blocking buffer for another 3 days. The antibody staining procedure was performed at 37°C with gentle shaking, combined with sonication at 40 kHz (60 w) for 2 hours each day (sonicator, LinVac 02ST). Samples were washed thoroughly with 1× PBST 3× for 1 hour after each antibody incubation, and a final wash was performed in 1× PBS for 2 hours after secondary antibody incubation, followed by 4% PFA post-fixation for 4 hours at room temperature and a thorough wash with 1× PBS 3× for 1 hour. Last, samples underwent a water dilation procedure for at least 1 day with gentle shaking at 37°C. Low-melting-temperature agarose water was used to maintain the rigidness of the expanded samples, as they were mostly water and became very fragile. Prior to imaging with lattice light-sheet microscopy, samples were transferred from agarose water to ddH_2_O at room temperature for at least 2 hours to avoid continuing expansion while imaging on a long-range XYZ Motorized Stage (MS-2000, Applied Scientific Instrumentation). Scanning Bessel beam light-sheet microscopy equipped with dual illumination arms and a long-working-distance multi-immersion objective (10×; numerical aperture [NA] 0.6; working distance [WD] 8 mm; Olympus, XLPLN10XSVMP) was controlled by the open-source software Micro-Manager (36). 3D image data of the whole kidney were acquired as multi-tile *z*-stacks at an image pixel resolution of 0.78 μm (*xy*) and *z*-step at 2~3 μm as sectioning interval. The data were further stitched by BigStitcher in Fiji/ImageJ (NIH) or Imaris Stitcher (Oxford Instruments) and visualized in 3D with Imaris (Oxford Instruments) or Amira (Thermo Fisher Scientific) (37, 38).

### Statistics.

Data analysis and curve fitting were performed with Prism (v6.07) software (GraphPad Software). Data are presented as mean ± SEM. Statistical comparisons between multiple groups of data were made using 1-way ANOVA with post hoc tests, 2-way ANOVA with mixed-effects analysis for body weight tracking, and 1-way repeated-measure ANOVA for water deprivation and DDAVP test. Comparisons between 2 groups were made using a 2-tailed unpaired *t* test. Survival curves for each group were compared using the log-rank test. Statistical significance was defined as *P* values less than 0.05. To track the TALs, the image raw data were downsampled to 10 μm/pixel in *xyz* dimension and tracked with the Cylinder Correlation module in Amira software (Thermo Fisher Scientific). The tracked TAL coordination was exported to MATLAB (MathWorks) for length analysis.

### Study approval.

All animal experiments were performed according to protocols (IACUC19-281, IACUC19-282) approved by the Laboratory Animal Center in the National Defense Medical Center, Taiwan.

## Author contributions

MHL, JCC, and YFL conducted experiments, acquired data, and analyzed data; XT, CML, and BCC carried out optical clearing and 3D imaging of mouse kidneys; ISY created Clc-k floxed mouse models; SU provided anti-Barttin antibody; CLH advised on the project and critically reviewed the data and revised the manuscript; CJC designed research studies, analyzed data, made the figures, and drafted and finalized the paper; all authors approved the final version of the manuscript.

## Supplementary Material

Supplemental data

Supplemental video 1

## Figures and Tables

**Table 1:**
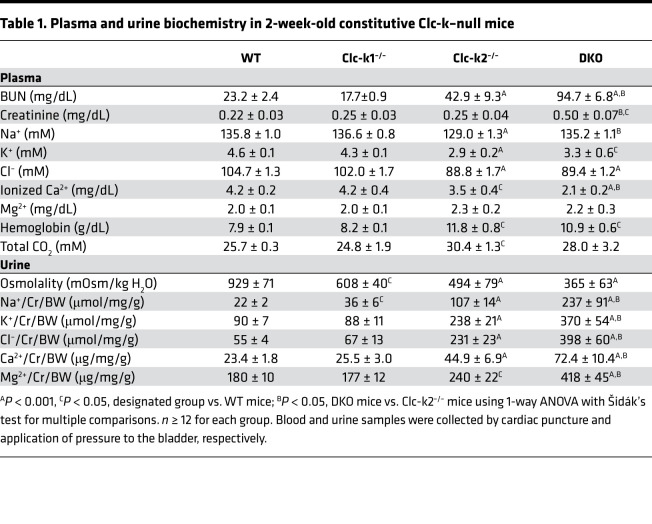
Plasma and urine biochemistry in 2-week-old constitutive Clc-k–null mice

**Table 2:**
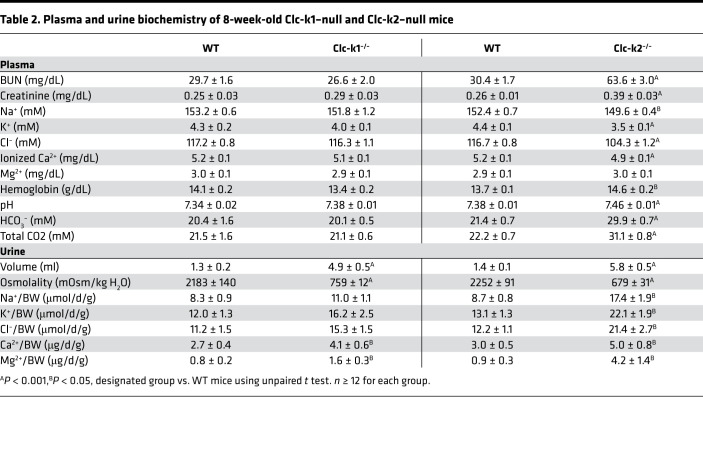
Plasma and urine biochemistry of 8-week-old Clc-k1–null and Clc-k2–null mice

**Table 3:**
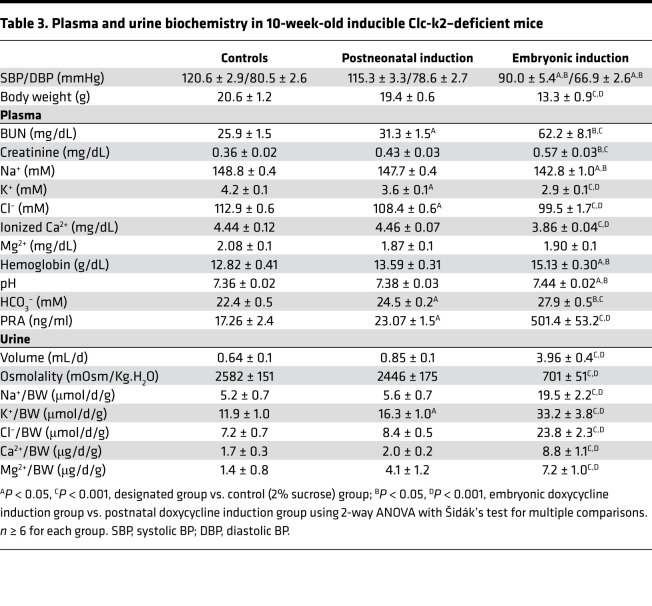
Plasma and urine biochemistry in 10-week-old inducible Clc-k2–deficient mice

## Version 1 09/09/2021

In-Press Preview

## Version 2 10/22/2021

Electronic publication
