# Supplementary material for: Impairment in renal medulla development underlies salt wasting in Clc-k2 channel deficiency
Source: JCI Insight. 2021 Oct 22;6(20):e151039. doi: 10.1172/jci.insight.151039 (PMC8564913; doi:10.1172/jci.insight.151039)
Supplement: Supplemental data [file jciinsight-6-151039-s136.pdf]

Impairment in renal medulla development underlies salt wasting in Clc-k2 channel deficiency

SUPPLEMENTAL MATERIALS

Table of Contents:

Supplemental methods

Supplemental Figure 1. Verification of the specificity of *Clcnk1* and *Clcnk2* RNA probes.

Supplemental Figure 2. Barttin expression in the P1-P7 developing mouse kidneys.

Supplemental Figure 3. Generation of *Clcnk1*$^{flox/flox}$, *Clcnk2*$^{flox/flox}$, *Clcnk1/k2*$^{flox/flox}$, and Ksp-Cre constitutive Clc-k knockout mice.

Supplemental Figure 4. Verification of the specificity of anti-Clc-k antibody against mouse Clc-k1 and Clc-k2.

Supplemental Figure 5. Quantitative measurements of *Clcnk1* and *Clcnk2* mRNAs in neonatal and adult Ksp-Cre/Clc-k knockout kidneys.

Supplemental Figure 6. Optic-clearing and the scheme for modified lattice light-sheet microscopy.

Supplemental Figure 7. Conditional Clc-k2 deletion after the neonatal period.

Supplemental Figure 8. The efficiency of Clc-k2 deletion in mouse models using constitutive Ksp-Cre or inducible Pax8-rtTA/LC1 system.

Supplemental video. Lattice light-sheet microscopy imaging of an optic-cleared, expanded, and Nkcc2-stained one-week-old mouse kidney.

Supplemental methods

Generation of *Clcnk1$^{flox/flox}$*, *Clcnk2$^{flox/flox}$*, and *Clcnk1/k2$^{flox/flox}$* mice using CRISPR-Cas9 method.

Cas9 mRNA, two single guide RNAs (sgRNA) targeting intron 2 and intron 15 of *Clcnk1* (intron 2 and intron 5 of *Clcnk2*), and two single-strand donor oligodeoxynucleotides (ssONDs) carrying a loxP site to be knocked-in at introns 2 and 15 of *Clcnk1* (intron 2 and 5 for *Clcnk2*), respectively, were co-injected into C57Bl/6 J mouse zygotes to generate mice with a floxed *Clcnk1* (at Exon 3 & 15) or a floxed *Clcnk2* (at Exon 3 & 5) allele via homology-directed repair. In short, the pX458 vectors expressing Cas9 and sgRNA targeting *Clcnk1* or *Clcnk2* genomic sequence were generated. The sgRNAs flanking *Clcnk1* or *Clcnk2* were designed using the CRISPR tool website (http://tools.genome-engineering.org) (Supplemental Figure 2). T7 promoter sequence was then added to the Cas9 coding region and to the sgRNAs by PCR amplification. T7-Cas9 and T7-sgRNA PCR products were used as templates for in vitro transcription with mMESSAGE mMACHINE T7 ULTRA kit (Thermo Fisher Scientific, MA, USA). Both the Cas9 mRNA and the sgRNAs were purified by MEGAclear kit (Thermo Fisher Scientific, MA, USA). Two ssODNs carrying a loxP site that was to be inserted into intron 2 or intron 15 of *Clcnk1* (intron 2 or 5 of *Clcnk2*) were designed (Supplemental Figure 3A, B). Purified Cas9 mRNA, sgRNA1, sgRNA2, 5' loxP OND, and 3' loxP ODN were co-injected into one-cell mouse zygotes (C57BL6/J) in M2 media (Millipore Corp. MA, USA) using a Piezo impact-driven micromanipulator. Post-injected blastocysts were transferred into the uterus of pseudopregnant female mice at 2.5 dpc. The preparation of mouse zygotes, pronuclei microinjection of Cas9 mRNA/sgRNAs, blastocysts transfer, and initial breeding of the *Clcnk1*$^{flox/flox}$ or *Clcnk2*$^{flox/flox}$ animals were performed by the Transgenic Mouse Core Laboratory in National Taiwan University. Because the floxed *Clcnk1* and *Clcnk2* genes could not be crossed into the same mouse due to the adjacency of the *Clcnk1* and *Clcnk2* genes, a loxP site was added into intron 2 of the *Clcnk2* gene in the floxed *Clcnk1* gene to flank both *Clcnk1* and *Clcnk2* genes (Supplemental Figure 3C).

PCR primers were designed to identify founders harboring two loxP knock-in sequences at the intended target site, as indicated by the presence of mutant PCR amplicons (Supplemental Figure 3D). The potential off-target mutageneses of CRISPR were assayed by RFLP/sequencing analysis at off-target sites predicted by CRISPR Design Tool. PCR, TA cloning, and DNA sequencing confirmed the presence

of an allele with successful knock-in of two lox-P sites that flanked exons 3 & 15 of *Clcnk1* (exon 3 & 5 of *Clcnk2*) in one of the founders. The founder was then backcrossing with wild-type C57BL6/J mice for three generations to dilute potential off-target. The F1 flox/+ progeny were then crossed to generate homozygous F2 flox/flox mice. F2 *Clcnk1*$^{flox/flox}$ and *Clcnk2*$^{flox/flox}$ mice were born at the expected Mendelian frequency and, at baseline, showed no detectable developmental defects or structural anomalies.

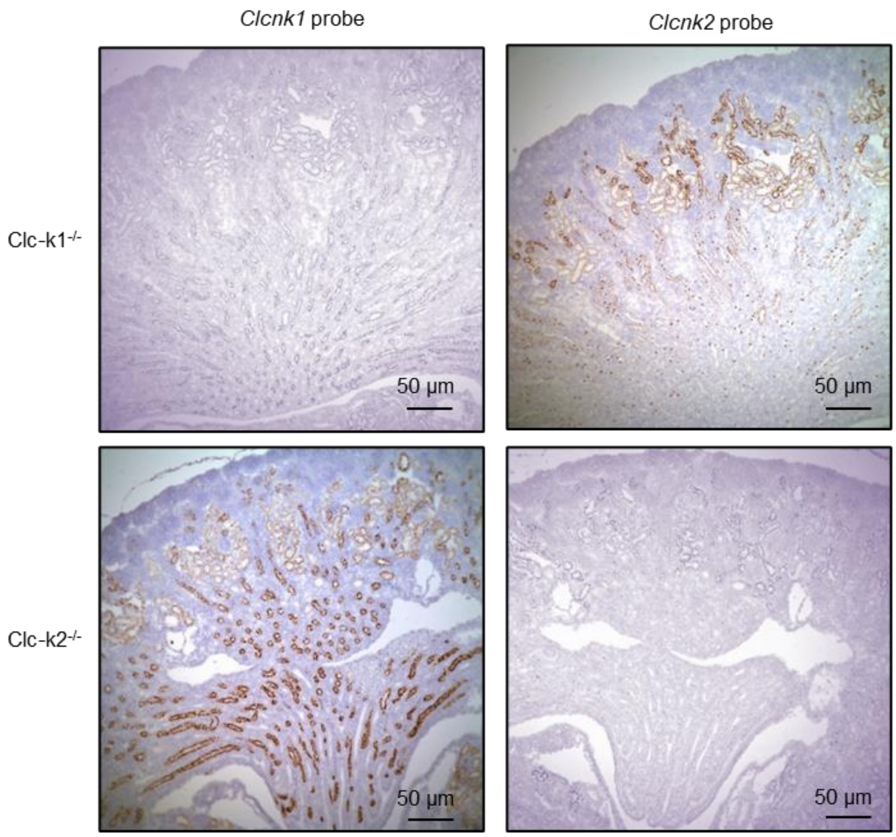

Supplemental Figure 1. Verification of the specificity of *Clcnk1* and *Clcnk2* RNA probes. *In situ* hybridization of *Clcnk1* and *Clcnk2* transcripts in one-week-old Ksp-Cre constitutive Clc-k1-null (Clc-k1$^{-/-}$) and Clc-k2-null (Clc-k2$^{-/-}$) kidneys using custom-designed *Clcnk1* or *Clcnk2* RNA probe. Trivial remaining signals in the kidneys of the knockout mice (left upper corner & right lower corner) could have resulted from the background created by the enzyme-based detection, rather than the cross-reaction between Clc-k1 and -k2 or ineffective gene deletion in knockout mice (the residual mRNA level of Clc-k1 and -k2 is less than 3% of wild-type controls).

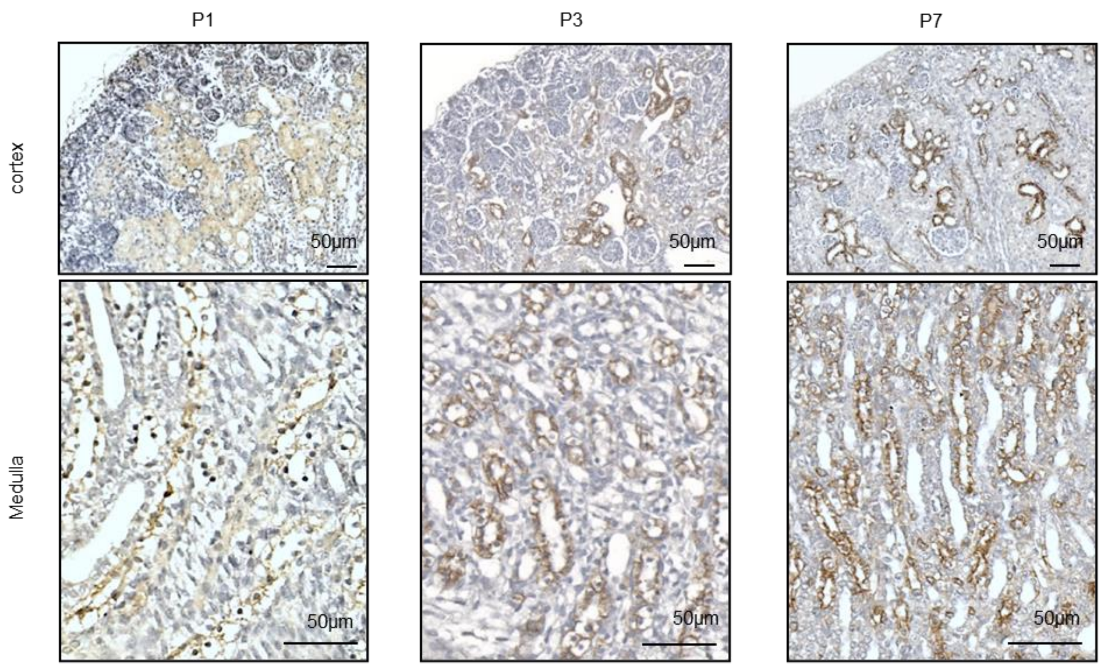

Supplemental Figure 2. Barttin expression in the P1-P7 developing mouse kidneys. Immunohistochemical staining of Barttin in wild-type neonatal kidneys.

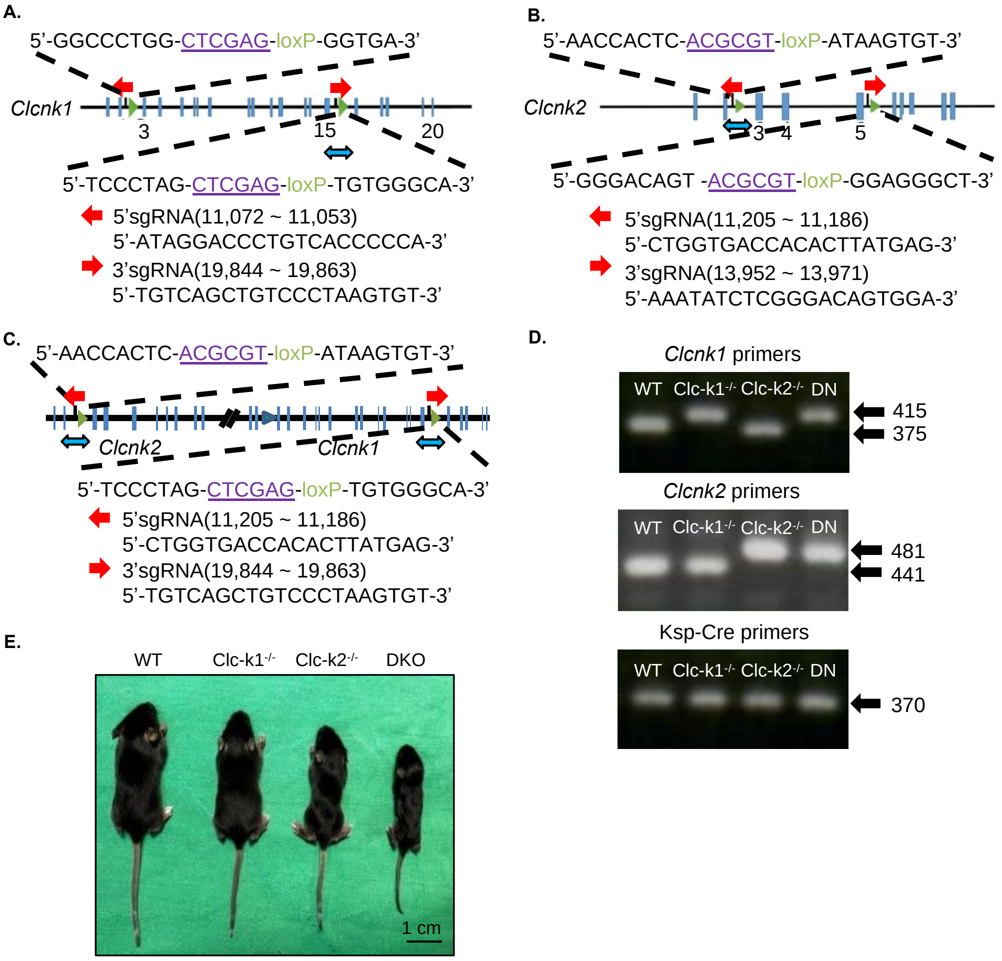

Supplemental Figure 3. Generation of *Clcnk1*$^{\text{flox/flox}}$, *Clcnk2*$^{\text{flox/flox}}$, *Clcnk1/k2*$^{\text{flox/flox}}$, and Ksp-Cre constitutive Clc-k knockout mice. (**A-C**) Sequences of sgRNA recognition sites (red arrows) and oligonucleotides used for loxP insertion (green arrowheads) are shown. For deleting both Clc-k1 and Clc-k2, a loxP site was added into intron 2 of the *Clcnk2* gene in the floxed *Clcnk1* gene. Double-headed arrows marked the locations of primers used for genotyping. (**D**) Genotyping of *Clcnk*$^{\text{flox/flox}}$ mice was carried out using PCR-based restriction analysis. The loxP containing 415-bp and 481-bp products were longer than the wild-type (WT) 375-bp and 441-bp products in the reactions using *Clcnk1* and *Clcnk2* primers, respectively. The PCR product for Ksp-Cre is 370-bp. (**E**) The gross appearance of 2-week-old WT and Ksp-Cre constitutive Clc-k knockout (Clc-k1$^{-/-}$: Clc-k1 knockout; Clc-k2$^{-/-}$: Clc-k2 knockout; DKO: Clc-k1/k2 double knockout) littermates.

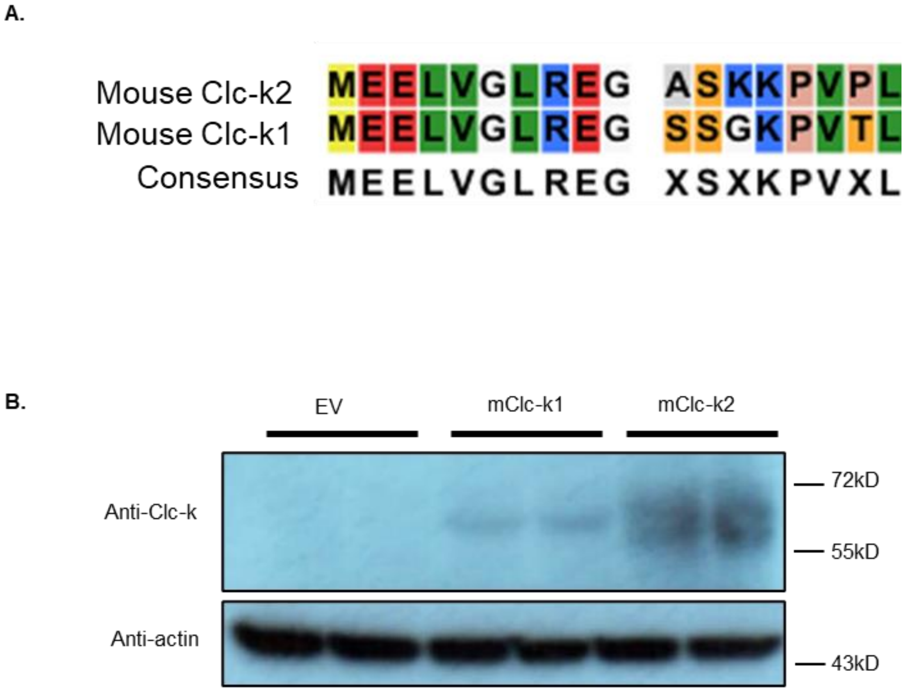

Supplemental Figure 4. **Verification of the specificity of anti-Clc-k antibody against mouse Clc-k1 and Clc-k2.** (**A**) Alignment of the first 18 amino acids in the N-terminus of mouse Clc-k1 and Clc-k2 proteins shows 83% homology in amino acid sequence. (**B**) The open reading frame of mouse *Clcnk1* and *Clcnk2* gene was cloned into pCMV6-entry (mClc-k1, mClc-k2 plasmids) for mammalian cell expression. An equal amount of protein extracted from HEK293 cells transfected with empty vector (EV), mClc-k1, or mClc-k2 plasmids was used for Western blot analysis against anti-Clc-k (1:1000 dilution) antibody.

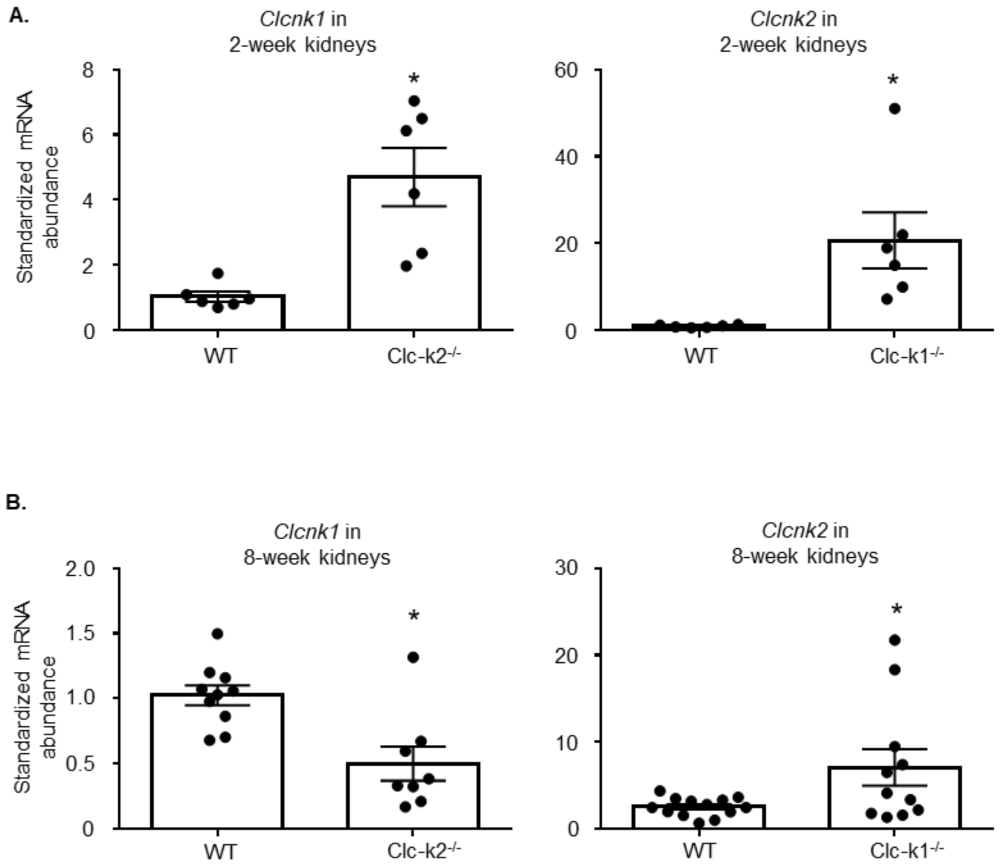

Supplemental Figure 5. Quantitative measurements of *Clcnk1* and *Clcnk2* mRNAs in neonatal and adult Ksp-Cre/Clc-k knockout kidneys. (**A, B**) The standardized mRNA levels of *Clcnk1* and *Clcnk2* in 2-week (**A**) and 8-week (**B**) whole-kidney lysates (n ≥ 6 for each group). *Clcnk1* and *Clcnk2* in each sample were standardized to the mRNA level of its own housekeeping *Gapdh* gene. The mean of *Clcnk* mRNA level in wild-type (WT) controls is set as 1. The comparison was performed by unpaired t-test. *$p < 0.05$ between designated group and WT controls.

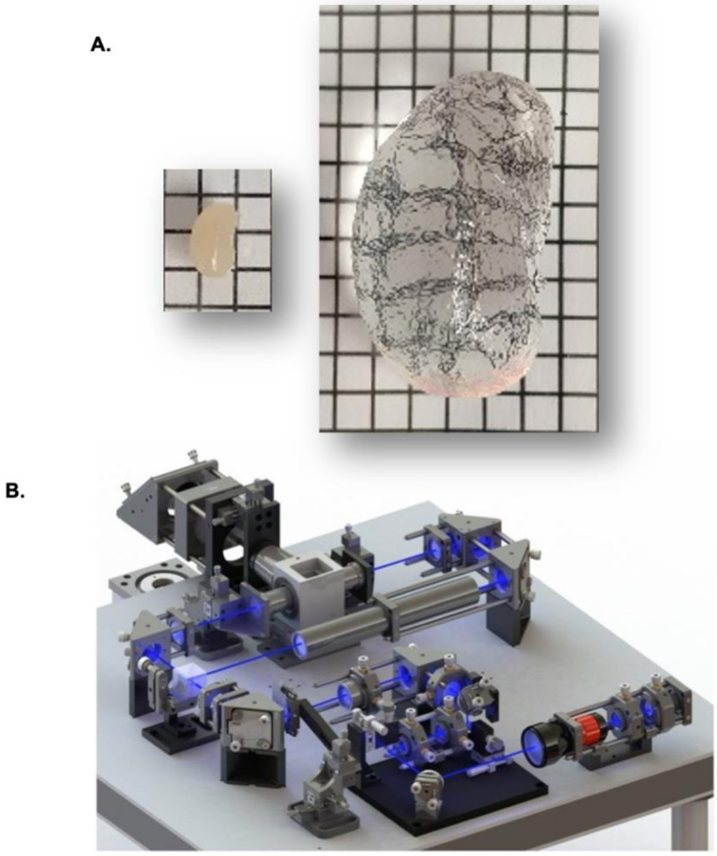

Supplemental Figure 6. Optic-clearing and the scheme for modified lattice light-sheet microscopy. (**A**) Optical clearing and expansion of one-week-old mouse kidney displays an isometric 4-fold expansion in all xyz axes. The same degree of expansion is tightly controlled in each sample. (**B**) Scanning Bessel beam light-sheet microscopy used for whole-kidney imaging.

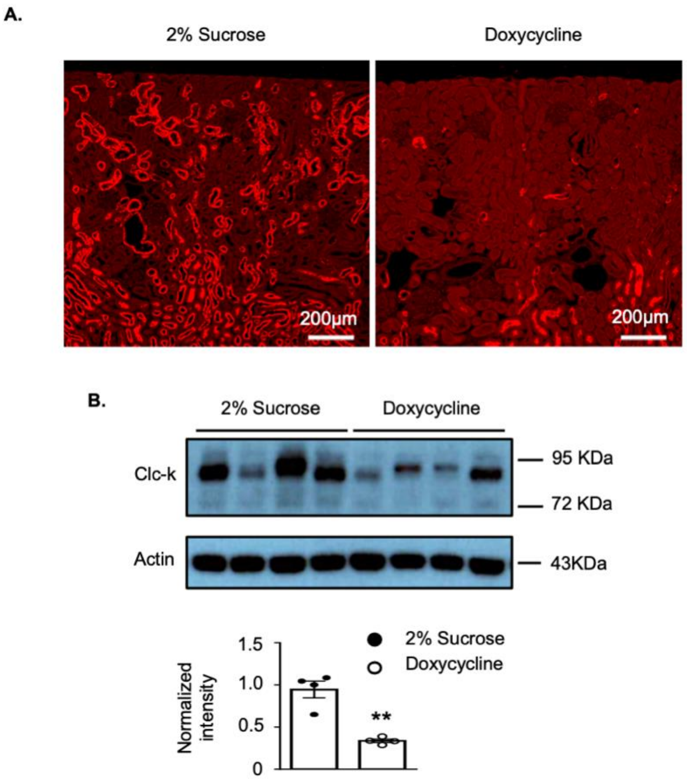

Supplemental Figure 7. Conditional Clc-k2 deletion after the neonatal period. (**A**) Immunofluorescence staining (red) and (**B**) Western blot analysis of Clc-k protein in doxycycline-induced Clc-k2 deficient kidneys (Doxycycline) and controls (2% Sucrose). The blots are representative of four similar experiments. **p < 0.001 between doxycycline-induced and control groups using unpaired t-test.

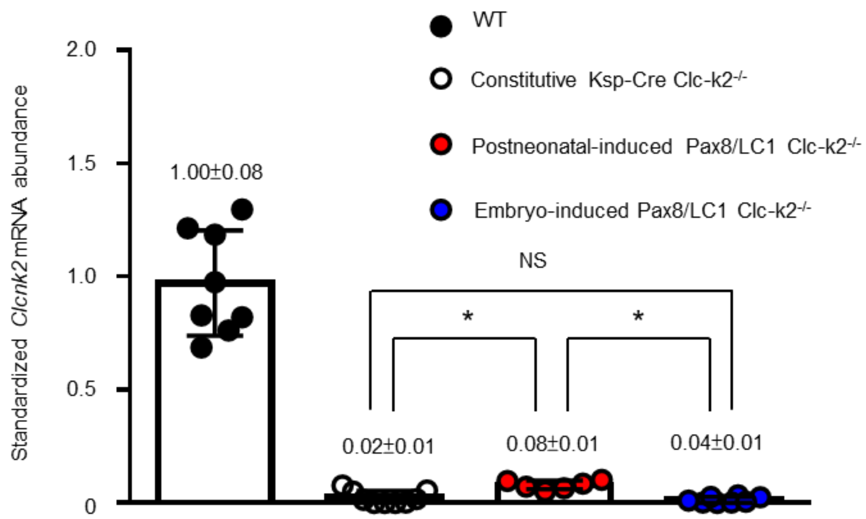

Supplemental Figure 8. The efficiency of Clc-k2 deletion in mouse models using constitutive Ksp-Cre or inducible Pax8-rtTA/LC1 system. Quantitative PCR analysis of Clc-k2 in 10-week-old wild-type (WT), Ksp-Cre constitutive Clc-k2$^{-/-}$, Embryo-induced Pax8/LC1 Clc-k2$^{-/-}$, and Postneonatal-induced Pax8/LC1 Clc-k2$^{-/-}$ mouse kidneys. *Clcnk2* mRNA transcripts in each sample were standardized to the mRNA level of its own housekeeping *Gapdh* gene. The mean of *Clcnk2* mRNA level in WT controls is set as 1.
